# Metallomic characterization of induced periapical lesions—In vivo study

**DOI:** 10.1111/iej.14274

**Published:** 2025-07-01

**Authors:** Jennifer Santos Pereira, Brenda Fornazaro Moraes, Anna Carolina Neves Leutz, Hellen Carolliny de Souza Nicolau, Rafaela Caires Santos, Talita Tartari, Brenda Paula Figueiredo de Almeida Gomes, Adriana de Jesus Soares, Ana Cristina Padilha Janini, Lauter Eston Pelepenko, Marina Angélica Marciano

**Affiliations:** ^1^ Division of Endodontics, Department of Restorative Dentistry, Piracicaba Dental School State University of Campinas (FOP‐Unicamp) Piracicaba SP Brazil

**Keywords:** endodontics, metal, metallomics, periapical lesion

## Abstract

**Aim:**

Metals are essential for metabolism and play a crucial role in various biological processes. Therefore, the aim of this study was to characterize and compare the metallomic profiles of periapical lesions and healthy alveolar bone in rats using complementary analytical techniques.

**Methodology:**

This study included 76 lower first molars (from 38 Wistar rats) where induced periapical lesions and controls (sham) were compared. Periapical lesion induction was performed by pulp exposure of these teeth, allowing infection development. After 40 days, the animals were reweighed, euthanised and their hemimandibles analysed by periapical radiography, histological analysis, micro‐computed tomography (μ‐CT), X‐ray fluorescence microscopy (μ‐XRF), scanning electron microscopy with energy dispersive spectroscopy (SEM/EDS), inductively coupled plasma optical emission spectrometry (ICP‐OES) and inductively coupled plasma mass spectrometry (ICP‐MS). Ten essential metals for metabolism were analysed (sodium, potassium, magnesium, calcium, iron, manganese, cobalt, copper, zinc and molybdenum). The analyses observed a significance level of 5%.

**Results:**

Radiographic analysis confirmed the induction of periapical lesions, without difference in animal weight between the conditions (*p* > .05). Histologically, the periapical lesions showed intense inflammatory infiltrate, cells with bluish cytoplasmic granules, alveolar resorption and scores ranging from moderate to intense. The μ‐CT analysis of the induced lesion revealed a significant difference in the periapical region volume (12.74 mm^3^, *p* = .0017). SEM/EDS showed limited sensitivity for the investigated chemical elements; however, μ‐XRF identified diminished intensities for calcium and zinc and increased intensities for iron. ICP‐MS and ICP‐OES identified increased concentrations of sodium (*p* = .0137), potassium (*p* = .0005), calcium (0.0059), magnesium (*p* = .0004), iron (*p* < .001), ^56^iron (*p* = .0078), ^57^iron (*p* = .0315) and manganese (*p* < .001) within the induced periapical lesion, suggesting a direct impact on mineral homeostasis following this pathology.

**Conclusions:**

This study demonstrated differences in the levels of various essential elements between conditions with periapical lesions and healthy controls.

## INTRODUCTION

Apical periodontitis is a chronic inflammatory disease caused by endodontic infection, with development regulated by the host's immune response (Gomes & Herrera, 2018; Nair, 2004; Sasaki et al., 2016). Initially, pro‐inflammatory macrophages are involved, whilst resolution involves macrophages and regulatory T cells promoting tissue repair. Symptomatic lesions are linked to pro‐inflammatory cytokines, RANK‐L (bone resorption), MMPs and ROS, contributing to tissue destruction and lesion progression (Hussein & Kishen, 2022).

Endodontic microbial communities in apical periodontitis have been studied in primary (Buonavoglia et al., 2023) and persistent lesions (Gomes et al., 2021). Treatment aims to disinfect the root canal, seal properly and rehabilitate the dental structure (Karamifar, 2020). Long‐term success depends on host–infection interaction, treatment effectiveness and tooth integrity maintenance (Gulabivala & Ng, 2023).

Primary endodontic treatment success in pulp necrosis and asymptomatic apical periodontitis is 81.1% (Da Silva et al., 2023) and non‐surgical treatments achieve 85%–94% success (Sjögren et al., 1997). Retreatment success rates are 74%–82% for non‐surgical (De Chevigny et al., 2008; Sundqvist et al., 1998), and 94% for microsurgery (Serefoglu et al., 2021), with mandibular first molars showing an 88% success rate (Serefoglu et al., 2021). These findings highlight the need for further investigation into factors beyond anatomy and microbiology.

The mechanisms of apical periodontitis involve macrophage polarization (Song et al., 2022), bacterial endotoxins (Lucisano et al., 2014), cytokine suppression (Braz‐Silva et al., 2019; Menezes et al., 2008) and diet influence (Tibúrcio‐Machado et al., 2021). Post‐treatment, bone remodelling mechanisms (Luo et al., 2022) and stem cell involvement (Lyu et al., 2022) aid in tissue repair (García et al., 2007).

No prior studies have analysed metallographic differences between healthy and apical periodontitis‐affected tissues. Evidence suggests incorporating metals into endodontic materials may modulate inflammation, stimulate bone repair and improve treatment predictability (Huang et al., 2021; Silingardi et al., 2024; Wu et al., 2020). This opens therapeutic possibilities for developing materials enriched with essential metals to promote tissue regeneration and repair.

Metallomics investigates the distribution, roles and dynamics of metal and metalloid species in biological systems (Roverso et al., 2023; Yasuda et al., 2020). The human body requires approximately 20 essential elements for proper metabolism, 10 of which (sodium, potassium, magnesium, calcium, iron, manganese, cobalt, copper, zinc and molybdenum) are metallic chemical elements (Jomova et al., 2022; Zoroddu et al., 2019). It is known that the catalytic behaviour of essential metabolic metals can lead to the formation of reactive hydroxyl radicals and oxidative stress, potentially causing damage to DNA, protein synthesis and membranes (Pizzino et al., 2017).

Changes in essential elements in biological tissues have been identified in various pathologies (Andrews, 2002; Bjorklund et al., 2018; Doroszkiewicz et al., 2023; Takeda, 2003), but evidence in apical periodontitis is lacking. Recent studies show that trace metal analysis can identify disease‐associated patterns (Stelling et al., 2019). For instance, thyroid diseases display unique metal profiles, including arsenic, lead, cadmium, copper, zinc and selenium (Stojsavljević et al., 2019). Altered zinc levels can impair antioxidant responses and worsen conditions like ischaemia/reperfusion (Smith et al., 2023). Pre‐eclampsia is associated with lower magnesium, calcium, iron, copper, zinc and selenium, with increased selenium potentially reducing the risk (Hao et al., 2024).

This study aimed to characterize and compare the metallomic profile of experimentally induced periapical lesions and healthy alveolar bone tissue in an animal model using multiple analytical approaches. Identifying specific metal patterns could improve the understanding of disease pathogenesis and unlock new perspectives for advancements in diagnosis, prognosis and more effective therapeutic strategies. The null hypothesis posits that there are no elemental differences between healthy and lesioned periapical bone.

## MATERIALS AND METHODS

**FIGURE 1:**
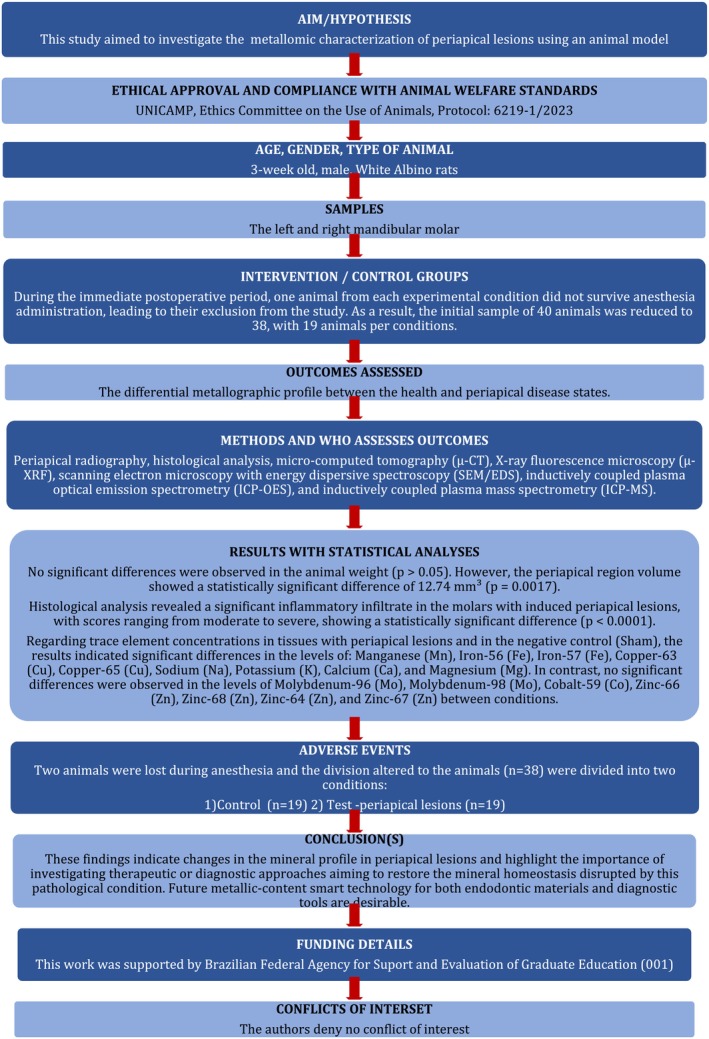
PRIASE 2021 flowchart illustrating the steps involved in conducting the present study.

This in vivo study was performed in accordance with the Preferred Reporting Items for Animal Studies (PRIASE) 2021 guidelines (Nagendrababu et al., 2020) (Figure 1), and following ethical guidelines approved by the Animal Use Ethics Committee—UNICAMP (Protocol CEUA: 6219‐1/2023).

### Sample size calculation

The sample size was determined using G*Power 3.0 software (Kavoli et al., 2017), considering a statistical power of 0.80, a 5% significance level and a medium effect size. The calculation resulted in 40 male *Rattus norvegicus*, Albinus lineage, Wistar strain (Table S1), of which two first mandibular molars, totalling 80 experimental units (*n* = 80), were desired.

Figure S1 shows the experimental design here further described.

### Animals

The animals were housed in appropriate facilities under the supervision of a veterinarian. Handling, feeding and monitoring were performed by an animal facility technician specialized in animal welfare. The animals were housed in an animal facility throughout the experimental period under a controlled room temperature of 22 ± 1°C and a 12‐h light–dark cycle (lights on from 7:00 AM to 7:00 PM). From birth until day 56 of life, the animals were kept in collective cages, having ad libitum access to water and food throughout the study.

### Induction of periapical lesion in lower molars

At 56 days of age, animals were weighed and anaesthetized using a combination of Ketamine (90 mg/kg) (Vet Brands Int, Miramar, Fl, USA) and Xylazine (10 mg/kg) (AnaSed®, Akorn Animal Health, United States) via intramuscular injection. After anaesthesia, the animals were carefully positioned on an operating table to allow for containment and mouth opening, providing access to the occlusal surface of the lower molars (Chicarelli et al., 2021; Kavoli et al., 2017). The mesial sulcus of the first lower molar was determined as the drilling point, as the root with a larger canal volume is anatomically located just beneath this structure.

In sequence, pulp exposure was performed bilaterally (Metzger et al., 2002) using a 1/2 spherical bur (EARC4, Dentsply Tulsa Dental Specialities, Oklahoma, United States), mounted on a high‐speed turbine. The bur was introduced to a depth of approximately 1 mm, avoiding furcation damage, with confirmation of pulp exposure by light probing and visual inspection of pulpal bleeding. To induce bacterial contamination, potentially leading to periapical lesion formation, teeth were left without sealing for a period of 40 days.

For analgesia, subcutaneous administration of metamizole sodium (100 mg/kg) (Neo‐Melubrina®, Sanofi, México) was performed immediately after the procedure, and it was also diluted in drinking water at a ratio of 200 mL of water to 0.2 mL of metamizole sodium. For the sham controls, a simulated intervention was performed, which included immobilization, anaesthesia and administration of metamizole sodium, but without pulp exposure procedure.

In the immediate postoperative period, one animal from each methodological condition did not survive the anaesthesia application, leading to its exclusion from the study. These animals were not replaced, thereby maintaining group pairing and consistency in the final sample (*n* = 76), comprising 38 animals with induced periapical lesions and 38 sham controls.

### Euthanasia of animals and sample preparation

After 40 days, all animals (92 days old) were reweighed using an electronic scale with an accuracy of 10^2^ g. Subsequently, a triple overdose of anaesthetic was administered, and ventral access to the heart was performed to ensure euthanasia and collection of biological samples. For analyses, the mandibles were carefully removed, dissected and divided into hemimandibles using blunt‐end scissors and a #15 scalpel blade (Bard‐Parker, Dickinson & Co., Franklin Lakes, USA). Samples were stored according to the specific methodological requirements. The experimental protocol for sampling and storage is shown in Figure S2.

The variation in sample size reflects the methodological requirements of each technique. Histological analysis (*n* = 9/group), being destructive, used specimens exclusively allocated for this purpose. μ‐CT (*n* = 9/group) was a non‐destructive technique. μ‐XRF and SEM/EDS (*n* = 10/group) were performed on the same samples, prepared to expose the periapical region. ICP‐OES (*n* = 9/group) and ICP‐MS (*n* = 8/group) used the same specimens at different dilutions; however, one sample was entirely consumed during ICP‐OES analysis, preventing its use in ICP‐MS.

### Analysis of periapical lesion area

Digital periapical radiographs were taken of all the hemimandibles (*n* = 76) using a radiology device (Dexis® Titanium, Dexis, United States). The hemimandibles were positioned perpendicular along the long axis of the tooth, with a focal‐film distance of 6 cm, 70 kVp and 7 mA parameters, and an exposure time of 0.3 s. The ImageJ software (version 1.54, National Institutes of Health, Washington, DC, USA) was used to measure the area suggestive of the induced periapical lesion.

For each image, the image calibration tool (set scale) was initially applied by entering the dimensions of the radiographic sensor (43 mm × 31 mm). Then, using the freehand selections tool, the radiolucent area at the apices of the first lower molar roots was manually outlined. The same operator took the measurement, and the area was obtained. When the image of the induced periapical lesion displayed fusion between the roots, a single area measurement was calculated, and when the lesions were separate, the area was measured individually for each root and these measurements were summed.

### Histological analysis

Histological analysis was conducted (*n* = 9 per group) in bone tissue blocks cut into a quadrangular shape, with the strategic removal of the condyles and incisor tooth to optimize processing. The specimens were placed in labelled cassettes and washed for 2 h under running water.

Over 80 days, the samples were immersed in 17% ethylenediaminetetraacetic acid (EDTA) with daily solution changes aiming for decalcification. Afterwards, these were embedded in paraffin and sectioned at 5‐μm thickness using a Leica RM 2155 microtome (Nussloch, Germany). The sections were then mounted on glass slides and stained with haematoxylin and eosin (H&E). Representative digital images of the periapical region, including bone tissue, dental root and soft tissue, were obtained using a Zeiss Axioskop II microscope (Switzerland), equipped with a Sony CCD IRIS RGB DXC‐151A camera (Tokyo, Japan) and Kontron KS300® software (München, Germany). Images were captured at 4×, 10× and 40× magnification for each histological section.

The intensity of periapical inflammation was evaluated using a scoring system. Manual counting of inflammatory cells was performed in six quadrants around the root apex by a single calibrated operator. The intensity of the inflammatory infiltrate was classified based on the mean cell count as follows (Aranha et al., 2013; Liu et al., 2012):

- Absent (0 to few cells)—Score 1;
- Mild (<25 cells)—Score 2;
- Moderate (25–125 cells)—Score 3;
- Severe (>125 cells)—Score 4.

### Micro‐computed tomography (μ‐CT) analysis of lesion volume

The hemimandibles (*n* = 9 per group) were dissected and stored at −20°C before analysis by μ‐CT. Scanning was performed using the SkyScan 1174 (Bruker, Kontich, Belgium) with a 0.5 mm aluminium filter, a pixel size of 6.46 μm, 360° rotation and a 1.0° step, with a total scanning time of 26 min, adjusted to a voltage of 55 kV and a current of 800 μA. Raw images were reconstructed using the NRecon software (Bruker, Kontich, Belgium) with a smoothing filter of 1%, beam hardening correction of 0%, ring artefact reduction of 1% and a grayscale dataset of 0.000–0.091. Subsequently, using the Data Viewer software (SkyScan, Version 1.4.4, 64‐bit), images were oriented and standardized in the three anatomical planes: transverse, longitudinal, and sagittal.

After reconstruction, the region of interest was defined using the CTAn software (v1.6.6.0, Bruker, Belgium) to include the bone resorption cavities of the entire sample under the first lower molars. The roots of the lower molars and the mandibular canals were excluded from the region of interest. This analysis was performed to quantify the periapical lesion volume (in mm^3^). The greyscale threshold was determined using a density histogram, generating a binary image with black and white pixels. For segmentation, a density histogram ranging from 21 to 255 was used to select bone tissue. Manual selection of the region of interest was performed in all image sets in the axial view, starting from the first image where all root apices of the left mandibular first molar were visible and ending 20 slices after the lesion disappeared (Figure S3). For the calculation of 2D areas and 3D volumes, the original greyscale images were processed with a Gaussian filter for noise reduction and an automatic segmentation threshold (Bouxsein et al., 2010).

### Fluorescence microscopy analysis (μ‐XRF)

For μ‐XRF and SEM/EDS (*n* = 10 per group), access to the periapical region was achieved through controlled wear using a Zekrya bur, ensuring uniform tissue exposure. In the periapical lesions, the wear was extended until the complete exposure of the lesion. A benchtop μ‐XRF system (Orbis PC EDAX, USA) equipped with a Rh anode operating at 45 kV and 200 μA was used. The equipment was configured to operate with a capillary optic of 30 μm. Detection was performed using a 30 mm^2^ silicon drift detector (140 eV FWHM at the 5.9 keV Mn‐Kα line). The pixels produced by the Orbis Vision software were linearly interpolated and mapped using Origin Lab 2016 software (Origin, Northampton, MA, USA).

### Scanning electron microscopy and energy dispersive X‐ray spectroscopy (SEM/EDS)

To perform a confirmatory analysis of the metallic content present in the periapical region, the same prepared samples were analysed using SEM/EDS. These were mounted on metal stubs and carbon coated. Photomicrographs were obtained in secondary electron mode using a scanning electron microscope (JEOL, JSM‐IT300, Akishima, Tokyo, Japan). Elemental mapping was conducted using the EDS line scan tool to determine the distribution of the elements of interest along the tooth–periapical region interface.

### Inductively coupled plasma mass spectrometry (ICP‐MS) and inductively coupled plasma optical emission spectroscopy (ICP‐OES)

Elemental quantification was performed using two spectrometry methods adapted to sensitivity requirements, including ICP‐OES (*n* = 9) and ICP‐MS (*n* = 8). Bone samples containing both the first mandibular molar and the periapical region with a weight between 15 and 22 mg were analysed. The samples were individually digested in Teflon rotors using an acid digestion process with 6 mL of 20% P.A. nitric acid (HNO_3_), redistilled by sub‐boiling, and 2 mL of 30% hydrogen peroxide (Sigma Aldrich—Merck, Germany). The containers were secured in the microwave rotor, and digestion was performed (Milestone, Shelton, CT, USA) under heating conditions: 1000 W power, 20 bar pressure, with a time/temperature cycle of 5 min at 160°C, 2 min at 160°C, 5 min at 170°C and 15 min at 170°C. After cooling to 60°C, the fully digested content was transferred to 15 mL Falcon™ tubes (Sigma‐Aldrich, Thermo Fisher, USA). Reagent blanks (without samples) were processed in each cycle for quality control.

For analysis, the samples were further transferred to new Falcon tubes (Sigma‐Aldrich, Thermo Fisher, USA) and diluted 1:10 (v/v) with ultrapure water (18 MΩ cm resistivity) from a Milli‐Q purification system (Millipore Sigma, Bedford, MA, USA). To ensure efficient removal of suspended solid particles, the samples were filtered using a 0.22 μm hydrophobic polyvinylidene fluoride syringe filter (Biocentrix, USA). Given the analysed elements, different isotopes were selected to achieve low detection limits. Quantification was performed using an ICP‐MS spectrometer (PlasmaQuant MS Elite, Analytik Jena, Jena, Germany) with an internal standard solution containing indium (In) (ICP‐grade, Merck, Darmstadt, Germany), antimony (Sb) (ICP‐grade, Merck, Darmstadt, Germany) and tin (Sn) (ICP‐grade, Chem Lab NV, Zedelgem, Belgium). Internal standards ranging from 0 to 50 ppb for molybdenum, cobalt, copper and manganese, and from 0 to 300 ppb for zinc and iron, were analysed alongside the samples.

Operational conditions for the elemental analysis performed using both spectrometers are presented in Tables S2 and S3. Calibration curves were constructed using multi‐element standards (1000 ± 3 μg/mL) diluted in 1% nitric acid (HPS, High Purity Standards, North Charleston, SC, USA). Additionally, standard laboratory rodent chow and wood shavings (bedding material) were collected and analysed via ICP‐OES for all the studied metals.

### Statistical analysis

Data were analysed using GraphPad 10.1.1 (323). Normality was assessed using the Shapiro–Wilk test. Depending on normality, comparisons between conditions were performed using either the *t*‐test or the Mann–Whitney test. A significance level of *p* < .05 was considered statistically significant. Graphical representations of statistical differences followed a specific convention: A bilaterally closed bar indicated a normal data distribution; a unilaterally closed bar signified that one dataset was normal (closed bar) whilst the other was non‐normal (open bar); and a bilaterally open bar denoted that both datasets were non‐normal.

## RESULTS

### Body weight assessment

Comparative analysis between animal conditions showed a normal distribution both initially (*p* = .4556) and after periapical lesion induction (*p* = .1904) (Figure S4).

### Radiographic analysis

The digital periapical radiographs (Figure S5) demonstrate the presence of induced periapical lesions. These lesions are notably characterized by well‐defined radiolucent areas surrounding the dental roots. In contrast, the sham control radiographs exhibit a continuous trabecular bone, and no evidence of bone rarefaction around the roots of the first mandibular molars.

### Histological analysis

**FIGURE 2:**
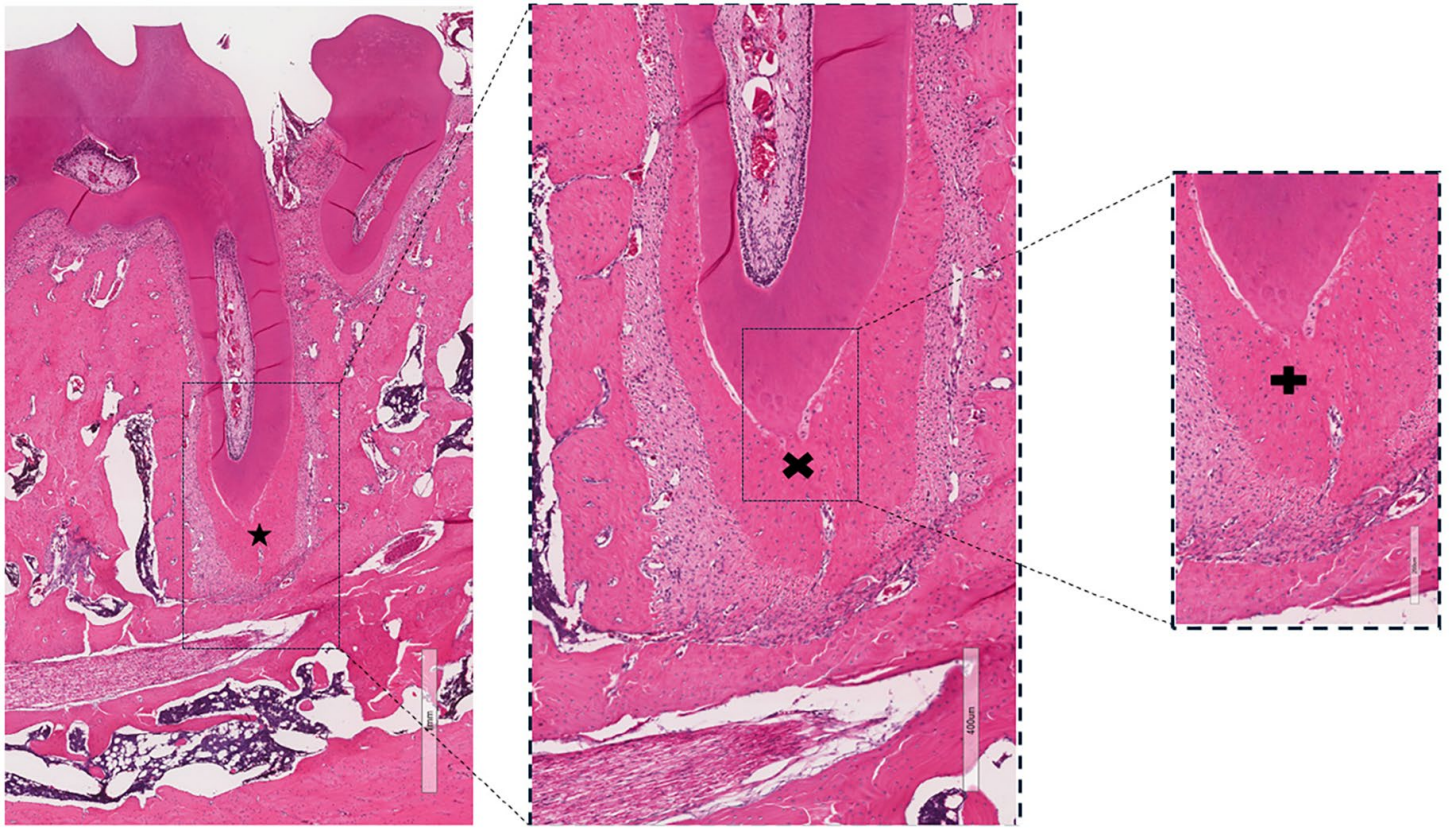
Histological image of bone tissue and dental structure under microscopic analysis, stained with haematoxylin and eosin (HE). The left image (scale: 1 mm) provides an overview of the tissue, highlighting the region of interest with a rectangle and marked by an asterisk (*). The intermediate magnification in the centre (scale: 400 μm) allows for a more detailed view of the cellular and structural characteristics of the delimited area, indicated by an ‘×’. The right image (scale: 200 μm) shows a higher magnification of the marked region, revealing specific details of the bone matrix and cellularity, indicated by the ‘+’ symbol. Scale bars are located at the bottom right of each image.

The histological sections of the molars from the sham controls showed no pulp or periapical tissue alterations (Figure 2).

**FIGURE 3:**
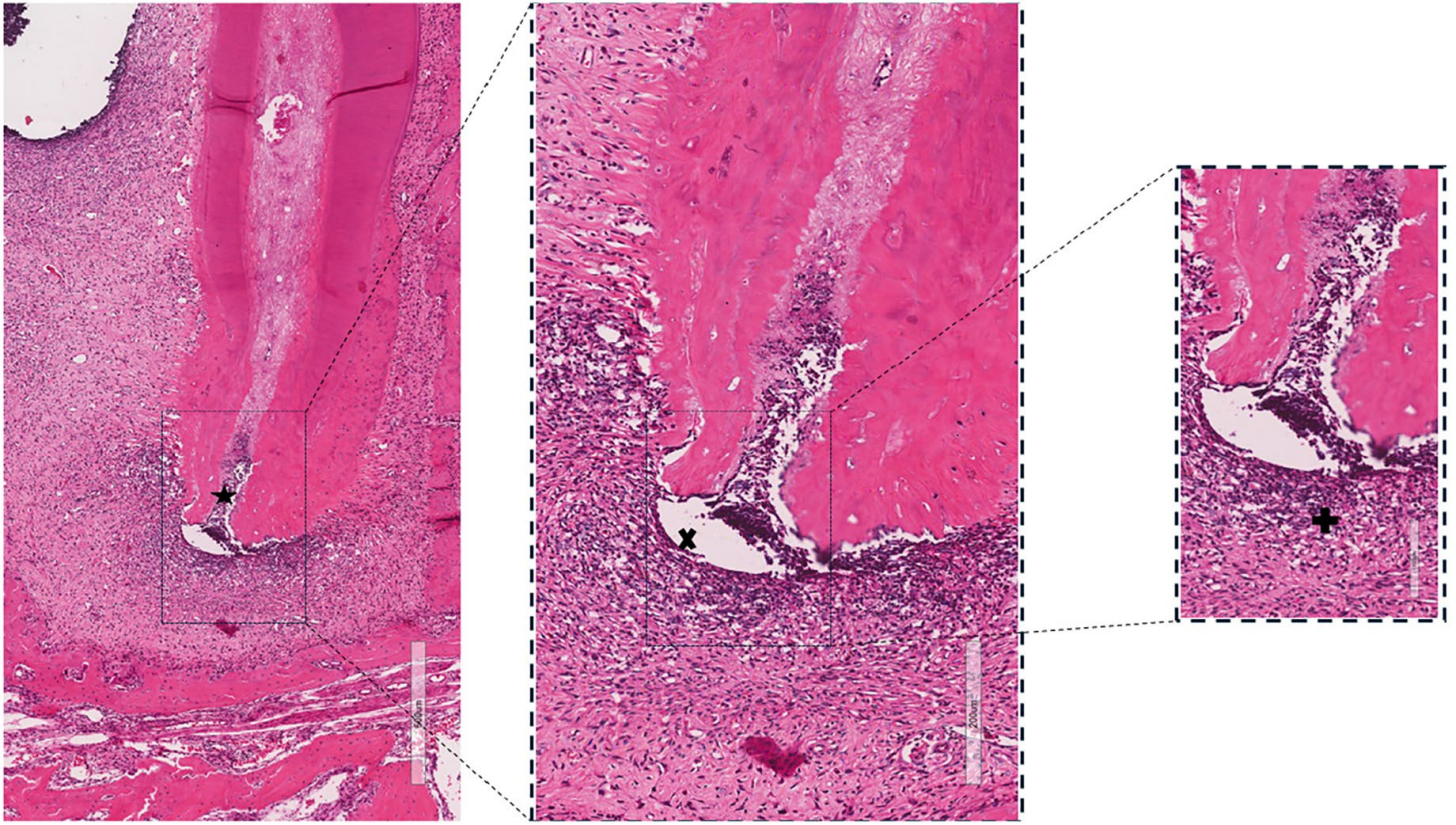
Light photomicrographs of the mesial root of the first mandibular molar with an induced periapical lesion. Images were obtained using 2×, 5×, and 20× objectives, with scale bars of 500, 200 and 100 μm in the left, middle and right panels, respectively. (*) Pulp necrosis. (×) Presence of a large abscess area, accompanied by resorption of bone, cementum and dentin. (+) Extensive periapical inflammatory infiltrate. The images highlight the morphological alterations resulting from inflammation and tissue destruction, emphasizing cellular organization and the interaction between the inflammatory infiltrate and mineralized tissues. Slides were stained with haematoxylin and eosin (H&E).

In molars with induced periapical lesions, the histological sections revealed an intense inflammatory infiltrate with neutrophils. Additionally, disorganization of the periodontal support structures was observed, in the mesial roots. A dense accumulation of polymorphonuclear leukocytes was also noted, with cells containing bluish cytoplasmic granules, areas of bone resorption and the presence of blood vessels. The observed characteristics are consistent with chronic periapical abscess formation. In the distal roots, inflammatory cells were sparsely distributed and restricted to the vicinity of the periapical foramen (Figure 3).

Teeth with induced periapical lesions did not have any samples classified with a score of 0. Three samples had a score of 1 (mild reaction), none had a score of 2 (moderate reaction) and six samples received a score of 3 (intense reaction), with a median score of 1.5. In the sham controls, the inflammatory infiltrate was classified as absent or with few inflammatory cells (score 0), with all nine samples receiving a score of 0. Statistical analysis showed a significant difference between conditions (*p* = .0001), highlighting the association between induced periapical lesions and a more intense inflammatory infiltrate.

### Micro‐computed tomography (μ‐CT)

**FIGURE 4:**
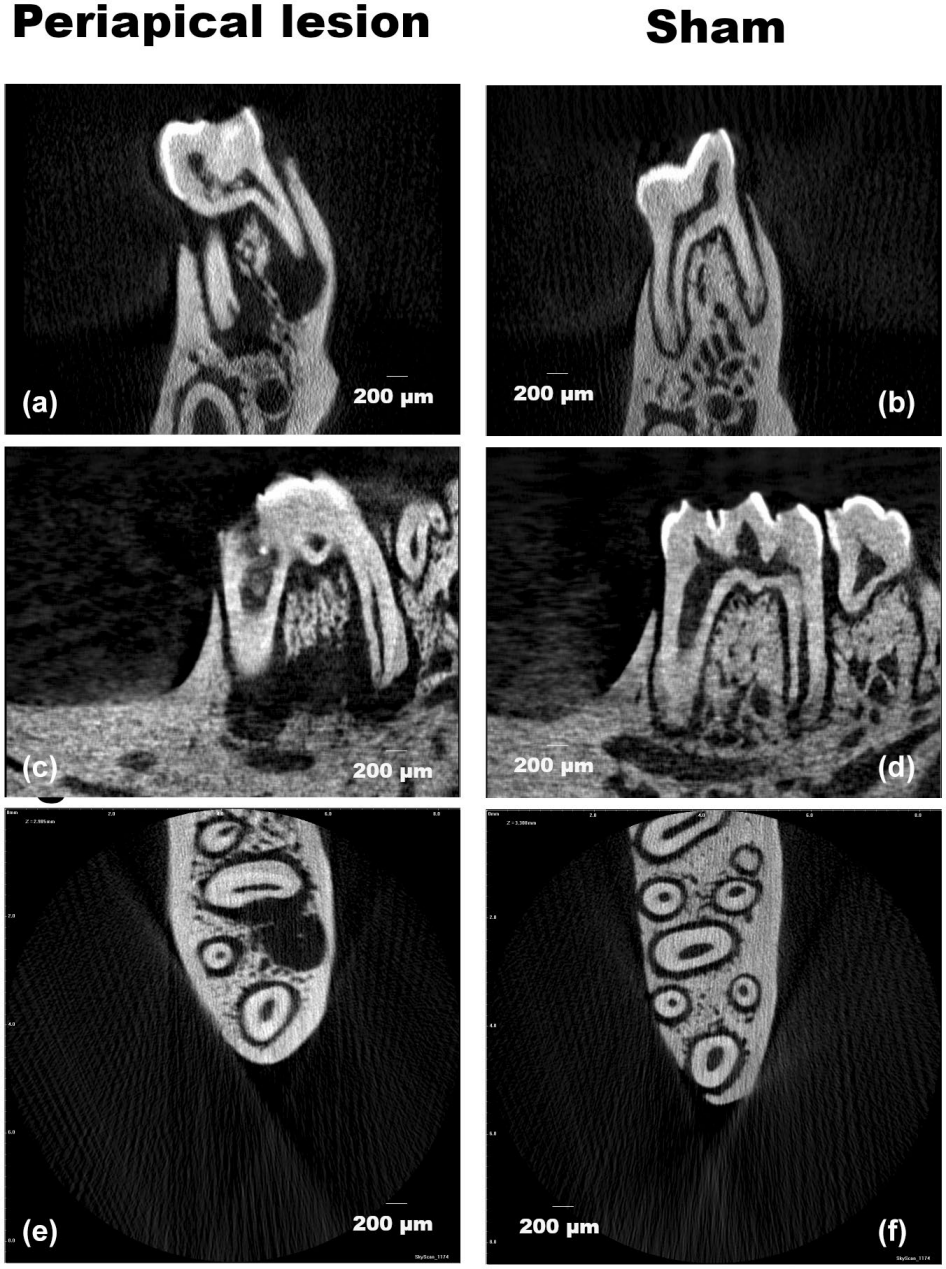
Micro‐computed tomography (μ‐CT) images of rat molars with induced periapical lesions (periapical lesion) and controls (sham). (a, b) Sagittal sections highlighting dental and bone morphology. In the induced periapical lesions, the extent and location of the lesion are observed, identified by a hypodense image in the apical region. In contrast, in the sham controls, the bone structure remains preserved. (c, d) Coronal sections focusing on the mesial region of the first mandibular molar, which was accessed for periapical lesion induction. In the induced periapical lesions, a hypodense shadow is noted in the furcation region, suggesting bone resorption. (e, f) Axial sections demonstrating, in the induced periapical lesions, a widening of the periodontal ligament space, accompanied by a hypodense image corresponding to the periapical lesion. In the sham controls, no loss of continuity is observed along the periodontal ligament space. The arrow indicates the periodontal ligament (f) and the white dot indicates the periapical lesion (e). Scale bars represent 100 μm.

This analysis indicated a statistically significant difference in the mean volume of the induced periapical region, measuring 12.74 mm^3^ (*p* = .0017). Figure 4 presents comparative images between conditions in sagittal, coronal and axial sections.

### Scanning electron microscopy/energy dispersive spectroscopy (SEM/EDS)

**FIGURE 5:**
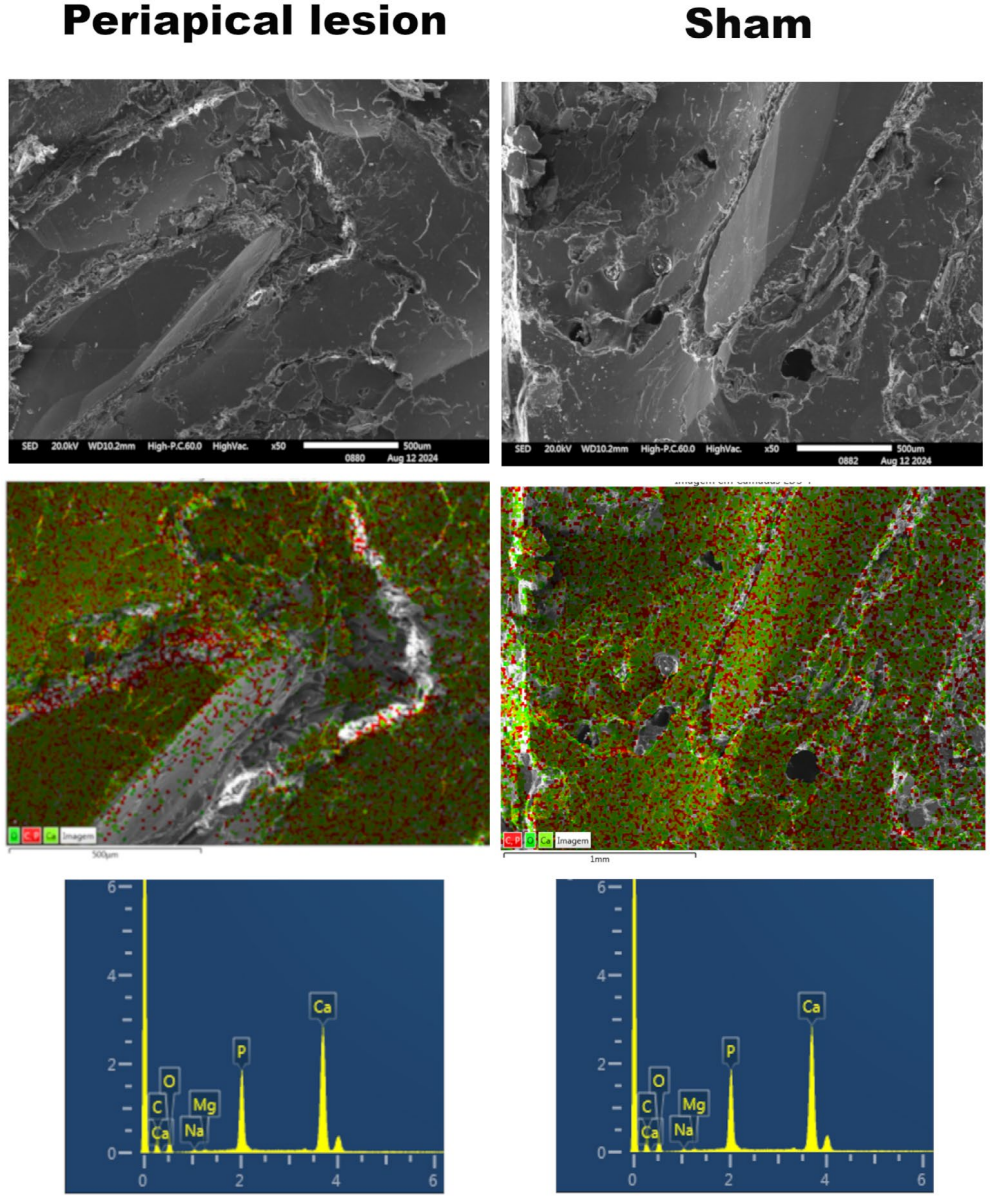
SEM/EDS analysis comparing periapical bone tissue between the periapical lesion and sham controls. (a, b) SEM images highlighting the microstructure of the bone tissue in a tooth with an induced periapical lesion, where the arrow indicates the periodontal ligament, the white dot indicates the periapical lesion and the white ‘X’ indicates the periapical root (a). In contrast, the sham controls (b) show preserved tissue, whilst areas of decalcification are evident in the induced periapical lesions. The scale bar represents 500 μm. (c, d) EDS elemental distribution maps, illustrating the distribution of calcium (Ca, red), phosphorus (P, green) and carbon (C, white) in teeth with periapical lesions (c) and sham controls (d). This analysis confirms the absence of calcium in specific areas within the periapical lesions. (e, f) EDS spectra highlighting the elemental composition of the samples, indicating the presence of oxygen (O), carbon (C), calcium (Ca), phosphorus (P), magnesium (Mg) and sodium (Na) in both conditions.

This analysis demonstrated morphological differences and variations in elemental composition (Figure 5). In the induced periapical lesions (a), areas of bone resorption, irregularities and lacunae were observed, suggesting structural loss. In contrast, the sham controls (b) exhibited a preserved surface without pathological defects.

Elemental analysis (c and d) revealed a reduction in calcium and phosphorus concentrations in the periapical lesion, indicating demineralization. Conversely, in the sham controls, the homogeneous distribution of these elements suggests the preservation of the bone mineral matrix. Additionally, variations in magnesium and sodium levels may reflect changes in the mineralization of the affected tissue.

However, this analysis provided limited results for a comprehensive characterization of the elements of interest, due to the lower sensitivity of the method. For this reason, it was essential to complement the findings with more robust techniques, such as μ‐XRF and ICP‐OES/ICP‐MS‐based methods, which offered greater accuracy and coverage in the identification and quantification of the investigated chemical elements.

### Fluorescence microscopy (μ‐XRF)

**FIGURE 6:**
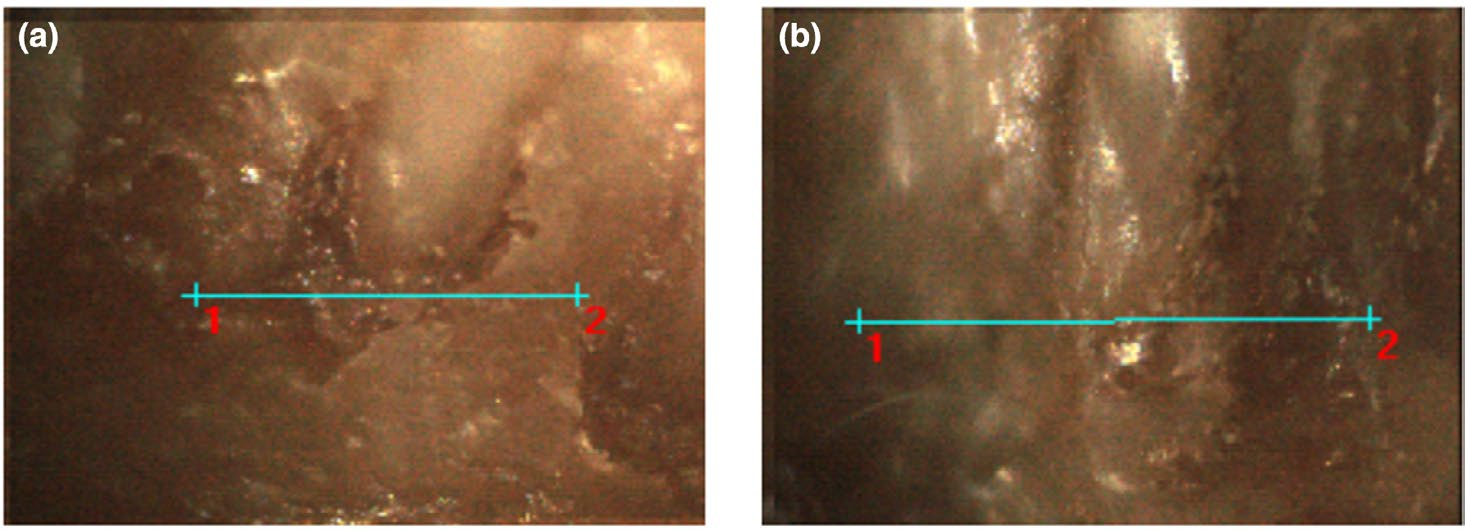
Highlight of the microscopic images ROI of hemimandibles from animals with induced periapical lesions and controls (Sham), analysed by μ‐XRF. In the induced periapical lesion (a) and the control (Sham) (b) is highlighted the periapical region where the linear scan was performed, indicated by a blue line (1.3 mm) with markings at the starting point (1) and the endpoint (2).

**FIGURE 7:**
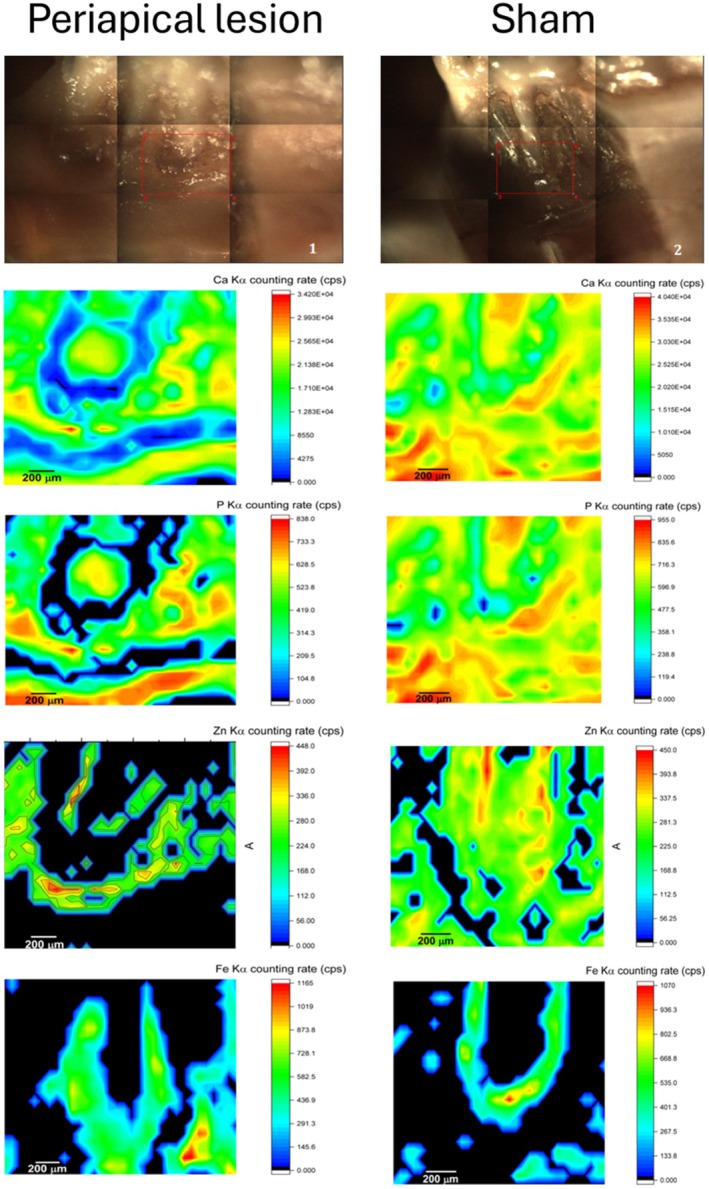
Comparative analysis between the periapical lesion and control (sham) conditions using μ‐XRF elemental distribution maps. Optical image of the analysed area in both conditions (1—Periapical Lesion and 2—Sham). The calcium (Ca Kα) map demonstrates a reduction in calcium intensity in the induced periapical lesions, indicating mineral loss, in contrast to the more homogeneous distribution in the sham controls. The phosphorus (P Kα) map corroborates this lower concentration in the periapical lesion, reflecting demineralization, whilst the concentration is maintained in the sham controls. The zinc (Zn Kα) map shows a more irregular distribution in the periapical lesion, whilst the sham controls exhibit a more uniform distribution. Finally, the iron (Fe Kα) map displays higher signal intensity in the induced periapical lesions, whilst in the sham controls, its levels are less intense.

In the elemental mapping analysis using μ‐XRF, the elements sodium, potassium, magnesium, calcium, iron, manganese, cobalt, copper, zinc and molybdenum were investigated. However, only three metallic elements (calcium, iron and zinc) were consistently detected in all samples, along with phosphorus, a non‐metallic element included in this analysis (highlighted in Figures 6 and 7).

Phosphorus, an essential component of calcium phosphate, is associated with calcium and plays a crucial role in mineral homeostasis. Its analysis—not initially aimed—was relevant for understanding alterations in elements related to bone health and mineralization, as well as evaluating interactions between metallic and non‐metallic elements in the context of lesions such as apical periodontitis.

Calcium exhibited low intensity in regions near the mesial root and the periapical lesion, whereas in the sham controls, it showed higher intensity and homogeneous distribution throughout the analysed region. Iron had lower intensity in the sham controls but showed variable intensity in the induced periapical lesions. Zinc was detected with higher intensity in the sham controls, whilst showing lower intensity in the lesion regions. Phosphorus also exhibited significant variations, with higher intensity in the sham controls and reduced levels in periapical lesion regions.

### Inductively coupled plasma mass spectrometry (ICP‐MS) and inductively coupled plasma optical emission spectrometry (ICP‐OES)

**FIGURE 8:**
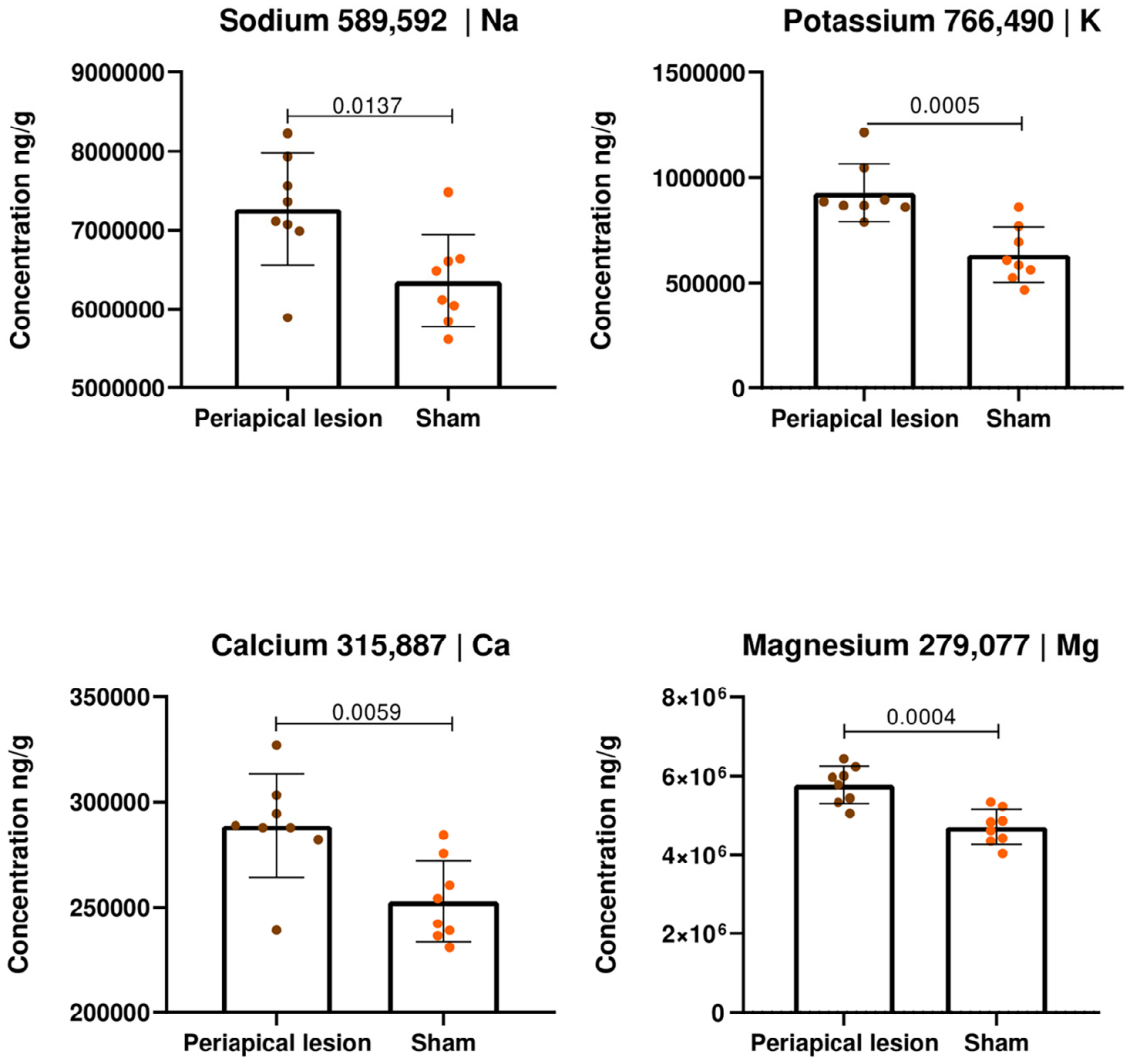
Graphical representation of the ICP‐OES analysis, showing the concentrations of essential elements (in ng/g) in tissues with periapical lesions compared to the control (sham). For these elements, significantly higher concentrations were observed within the periapical lesions.

**FIGURE 9:**
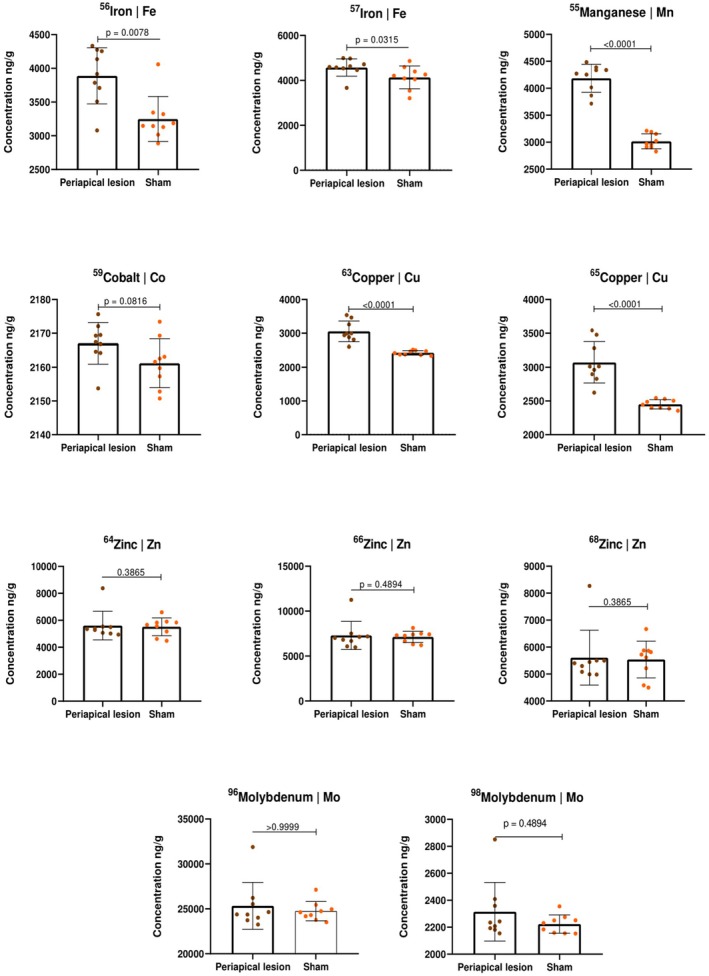
Graphical representation of the ICP‐MS analysis showing the concentrations (ng/g) of different elements in tissues with periapical lesions compared to the control (sham). No differences were observed between the isotopes in terms of statistical significance.

The concentrations of sodium, potassium, calcium and magnesium were measured using ICP‐OES (Figure 8), whilst iron, manganese, cobalt, copper, zinc and molybdenum were measured by ICP‐MS (Figure 9) after acid digestion.

The elemental concentration analysis by ICP‐OES revealed statistically significant differences between the periapical lesion and sham controls for sodium, potassium, calcium and magnesium. Sodium showed significantly higher concentrations in the periapical lesions (approximately 7 500 000 ng/g) compared to the sham controls (approximately 6 000 000 ng/g) with *p* = .0137. Similarly, potassium levels were higher in the periapical lesions (1 000 000 ng/g) compared to the sham controls (600 000 ng/g) with *p* = .0005. Calcium also exhibited higher concentrations in the periapical lesions (300 000 ng/g) compared to the sham controls (250 000 ng/g) with *p* = .0059. Finally, magnesium showed a significant increase in the periapical lesions (6 000 000 ng/g) compared to the sham controls (4 500 000 ng/g) with *p* = .0004.

The elemental concentration analysis by ICP‐MS revealed statistically significant differences (*p* < .05) in the iron isotopes between the sham controls and induced periapical lesions, with higher concentrations in the latter. Similarly, manganese and copper levels were significantly higher in the periapical lesions (*p* < .0001). However, no significant differences were observed for the zinc, cobalt and molybdenum isotopes (*p* > .05). In general, a higher metallic content was observed within periapical lesions when compared with sham controls, regardless of the metal analysed.

### Analysis of feed and wood shavings

The feed showed detectable concentrations of sodium (34 730 ng/g), magnesium (54 086.67 ng/g), potassium (170 033.33 ng/g), calcium (82 103.33 ng/g), iron (3061.33 ng/g), copper (20 ng/g) and zinc (673 ng/g). In contrast, the wood shavings showed no detectable concentrations of these elements, except for sodium (7 ng/g).

It is noteworthy that all animals were kept under the same housekeeping conditions.

## DISCUSSION

The experimental induction of periapical lesions can be performed using various methods, including pulp exposure (Brilhante Wolle et al., 2012; Liu et al., 2012) or stimulation with lipopolysaccharides and other endodontic pathogens (Aranha et al., 2013; Fukada et al., 2008). In this study, pulp exposure to the oral environment during the experimental period was chosen, aiming to simulate a clinical condition of microbial contamination. This methodology was able to promote periapical lesions, inducing an inflammatory reaction similar to that observed in other studies (Almeida et al., 2024; Anan et al., 1993; Silva et al., 2011; Zhang & Peng, 2005). For the induction of apical periodontitis lesions, the first lower molar was selected due to its anatomical similarity to human teeth (Dammaschke, 2010) and the biological progression of the inflammatory response in rats (Moretton et al., 2000) within the experimental timeline here proposed.

The analysis of body weight showed that bilateral pulp necrosis and periapical lesion did not significantly affect this criterion (*p* > .05). This suggests that the local inflammation did not cause a significant systemic impact on the animals' weight. A possible recommendation of unilateral induction to avoid influencing animal weight could be refuted by the present study since a bilateral induction was here performed. A previous study (Şehirli et al., 2024) indicated apical bone resorption following induction solely on pulp chambers of the right mandibular first molar, also with an increase in periapical radiolucency, as here observed bilaterally.

Periapical bone resorption was here analysed using different methods, such as histological evaluation, digital radiography and μ‐CT, following methodological approach as previous studies (Rittling et al., 2010; Sun et al., 2017; Teixeira et al., 2011). Additionally, the present study conducted observations under SEM, which revealed the periapical lesion and allowed for sample mapping through EDS. This methodological addition was important to validate the expected calcium deficiency in the periapical lesion area and, along with phosphorus, demonstrated the absence of bone tissue observed in periapical lesions. However, the further addition of μ‐XRF and ICPs were essential to deepen the elemental analysis of metals here desired.

The area analysis allowed for the identification of visible differences between the left and right sides of the periapical lesions. However, conventional radiographs often underestimate the actual size of periapical lesions (Ferreira et al., 2006), as they do not allow for an accurate assessment of the lesion's extent. Periapical lesions only become evident when there is involvement of the cortical plate or junctional trabeculae. This finding is consistent with previous studies (Attaelmanan et al., 2000; Hamachi et al., 1995; Yoo et al., 2023).

The μ‐CT analysis allowed for the identification of the average lesion volume of 12.74 ± 3.02 mm^3^ (standard deviation), aligning with previous reports that observed volumes of 12.15 mm^3^ after 4 weeks. This expansion occurs more rapidly during the first 15 days (active phase) and slows down thereafter (chronic phase) (Okiji et al., 1994; Stashenko & Yu, 1989; Wang et al., 2009; Wang & Stashenko, 1991; Yoneda et al., 2017). The decrease in periapical lesion volume after 4 weeks reinforces the hypothesis of a modulation of the host immune response against bacterial infection during this period (Yoneda et al., 2017).

The experimental period of 40 days was here chosen to ensure that the induced periapical lesions reached a sufficiently advanced stage, allowing for a clearer detection of pathological alterations. This period was ideal for observing changes in the periapical structure here observed and ensure that the lesions were well developed for analysis, consistently with previous findings (Armada‐Dias et al., 2006; Brasil et al., 2017, 2021).

In the histological sections of the molars from the sham controls, the analyses did not reveal significant alterations in the pulp and periapical tissues, highlighting healthy and well‐preserved structures. The integrity of these tissues suggests the absence of harmful stimuli or inflammatory processes, establishing a crucial baseline for comparison with animals with induced periapical lesions. In contrast, in the molars with induced periapical lesions, the histological findings revealed an intense inflammatory response, particularly concentrated in the apical region of the root. This process could be entirely attributed to pulp exposure, which allowed bacterial colonization and proliferation within the pulp tissue, followed by its necrosis and subsequent infection of the periapical tissues. Bacterial infection triggers neutrophil activation, leading to a massive migration of these cells to the apical foramen (Yamasaki et al., 2008). In this study, dense accumulations of polymorphonuclear leukocytes containing bluish cytoplasmic granules associated with blood vessels were identified. These findings align with previous reports describing the formation of periapical abscesses 3 weeks after pulp exposure (Matsui et al., 2011).

In this area of bone tissue resorption, the further analyses (SEM/EDS, μ‐XRF, ICP‐OES and ICP‐MS) were performed, revealing significant differences in the metallographic profile when comparing healthy bone tissue with that affected by apical periodontitis. This indicates that the changes were not limited to decalcification altering the calcium metallographic profile.

Previous studies emphasizes the fundamental role of essential metals in regulating physiological processes, ensuring the balance of vital functions in the body (Baj et al., 2023; Costa et al., 2024). Imbalances in the homeostasis of these elements can induce cellular stress and promote disease development (Baj et al., 2023; Cannas et al., 2020; Ceko et al., 2016; Himoto & Masaki, 2020; Kamińska et al., 2021; Xu et al., 2023). Given the limited availability of comparative studies focusing on the composition and distribution of these metals in an endodontic context, direct comparisons with existing literature remain challenging.

Given the complexity of identifying and quantifying these elements, this study utilized ICP‐MS and ICP‐OES techniques to determine their concentrations between conditions. Amongst the analysed metallic elements, seven of them (sodium, potassium, magnesium, calcium, iron, manganese and copper) showed significant differences between conditions, all with higher concentrations in the periapical lesions. The homeostasis of healthy bone tissue is maintained essentially by osteoblasts, osteoclasts and osteocytes, as well as by an organic matrix (mainly type I collagen) and inorganic minerals (apatites), primarily hydroxyapatite (Boivin & Meunier, 2003; Henmi et al., 2017). This complex composition grants bone tissue its dynamic properties, allowing constant metabolic interactions between the chemical elements within the extracellular fluid and the apatite crystals that form the mineral matrix (Boivin & Meunier, 2003). Not surprisingly, elemental exchanges involving magnesium, zinc and strontium for calcium, as well as carbonate for phosphate, have been associated with normal bone metabolism (Maciejewska et al., 2014).

Intuitively, a reduction in calcium concentration in the periapical lesion is expected, as bone resorption associated with the inflammatory process tends to result in mineral loss. Calcium depletion in the cellular environment has been associated with a significant reduction in the mineralization rate (Bellows et al., 1991). The μ‐XRF analysis corroborated this expectation, demonstrating a lower calcium signal intensity in the affected periapical region compared to the healthy condition, where the signal was significantly stronger. The same pattern was observed for phosphorus, which showed a reduced or nearly absent signal in the lesion, whilst remaining elevated in the healthy condition. However, the results obtained by ICP‐OES indicated a significantly higher total calcium concentration in the induced periapical lesions compared to the healthy condition. This apparent contradiction can be partially explained by the differences in the measurement principles of the techniques. ICP‐OES quantifies the total concentration of dissolved calcium in the sample without distinguishing its spatial distribution (bone matrix, extracellular phase or protein‐associated fraction) (Szymczycha‐Madeja et al., 2021), whereas μ‐XRF provides a surface mapping of up to 20 μm of the element's location (Maciejewska et al., 2014). Therefore, it is not possible to obtain the depth distribution of these elements using these techniques alone.

Further discussion on the calcium content can be established based on suggestions that bone regeneration is a complex process involving inflammation, induction and remodelling, intrinsically regulated by the substantial interaction between innate immune cells and bone cells (Jeong et al., 2025). Calcium is recruited to the bone defect site through a process in which osteoblasts are attracted to the lesion area by chemical signals, leading them to migrate and deposit minerals, effectively initiating bone regeneration. Whilst inflammation is an essential part of the bone regeneration process, a chronic or persistently dysregulated inflammatory response in inflammatory alveolar disease is detrimental to the surrounding tissues (Hussein & Kishen, 2022). This process can be further enhanced by the use of biomaterials such as calcium phosphate ceramics, which serve as a scaffold for calcium deposition and cell adhesion, essentially mimicking the natural bone matrix (Amini et al., 2012).

Additionally, the relationship between calcium and phosphorus must be considered. Phosphorus, in the form of phosphate, is known to inhibit both active cellular resorption and bone mineral dissolution (Raisz, 1969). However, in this study, its concentration was found to be reduced in the periapical lesion, which may suggest that, under certain pathological conditions, this inhibition of bone resorption does not occur effectively. This decrease in phosphate availability may be associated with an imbalance in local bone metabolism (Tenenbaum et al., 1989), favouring disorganized calcium deposition or serving as a response to the inflammatory process (Sigel et al., 2013). Thus, the findings of this study indicate that, despite the characteristic bone resorption of periapical lesions, the higher calcium concentration detected by ICP‐OES may reflect the presence of reactive calcification, remnants of mineralized matrix or even an initial bone repair process, representing an attempt by the body to prevent the progression of the periapical lesion.

Although iron concentration was significantly higher in the induced periapical lesion conditions compared to the sham control, its distribution, as observed in the μ‐XRF mapping, was limited to the apex of the periapical region and the adjacent bone tissue. This localized distribution was not observed in the healthy bones. In the ICP‐MS analysis, the concentrations of the isotopes ^56^iron and ^57^iron were investigated, as they are the most abundant, whilst ^54^iron is considered potentially radioactive (Dauphas & Rouxel, 2006). The results revealed statistically significant differences between the experimental conditions and may be related to the essential role of iron in collagen biosynthesis (Beattie & Avenell, 1992), in the formation of the bone matrix (Maciejewska et al., 2014) and its significant influence on bone mineral density (Medeiros et al., 2002).

Both manganese and copper were elements that showed an increased concentration in the induced periapical lesions. Copper has two predominant and stable isotopes (Lauwens et al., 2018), whilst manganese has only one stable isotope (Moreira et al., 2024). Both metals possess antioxidant properties, which may be associated with an increased need for these metals to neutralize free radicals and protect cells (Chellan & Sadler, 2015; Lowe et al., 2002). Copper's effects are closely related to iron homeostasis and the regulation of reactive oxygen species (Yu et al., 2024). Manganese, on the other hand, plays a crucial role in the synthesis of mucopolysaccharides (Saltman & Strause, 1993), and its deficiency is associated with impairments that delay the osteogenesis process (Cashman & Flynn, 1998). This element also appears to play a role in the regulation of bone remodelling, as its absence has been previously correlated with elevated extracellular concentrations of calcium, phosphate and alkaline phosphatase (Bergstrom, 1954; Friedman et al., 1987).

Both sodium and potassium showed increased concentrations with significant differences between conditions, highlighting the potential impact of local inflammatory lesions on the concentration of these elements in bone tissue. This finding supports a previous study (Starke et al., 2012) that describes how potassium and sodium play a crucial role in bone health, modulating the activity of osteoblasts and osteoclasts through potassium and sodium channels present in the cell membranes of these cells. The presence and proper functioning of these channels are essential for the homeostasis of the bone microenvironment, as they regulate the intracellular chemical balance, which is crucial for bone formation and resorption processes (Singh & Kushwaha, 2024). Thus, potassium and sodium not only act as essential electrolytes for systemic functions (Sigel et al., 2013), but also play a specific role in bone health (Bergstrom, 1954).

Magnesium is an essential cofactor in many biochemical reactions that occur in bone (De Baaij et al., 2015), especially in the formation and maintenance of hydroxyapatite (the main mineral form of bone), as it helps stabilize hydroxyapatite crystals and, therefore, is important for the structural integrity of bone (Salimi et al., 1985). The results showed a statistically significant difference with an increase in magnesium concentration when comparing animals with induced periapical lesions to the control (sham). This alteration may be related to the magnesium regulatory role in inflammation, as altered levels of this mineral influence the release of pro‐inflammatory cytokines and modulate macrophage activity, which are essential cells in the immune response to the infectious process (Maier et al., 2021).

Although zinc did not show a statistically significant difference between the healthy and diseased states, it has been reported that this metal plays a role in the growth, development and maintenance of healthy bones by promoting osteoblast activity, inhibiting osteoclast function and stimulating the synthesis of bone proteins, which results in increased bone mass and growth (Bouglé et al., 2004; Fang et al., 2024; Yamaguchi et al., 2000). The μ‐XRF analysis (by mappings) revealed that the distribution of zinc is not homogeneous in both conditions, health and disease. A greater number of areas with reddish coloration, indicative of elevated zinc concentrations, were observed in the sham control compared to the induced periapical lesions. However, the ICP‐MS analysis did not reveal statistically significant differences in zinc concentrations, even when considering the different isotopes of this element (Cloquet et al., 2008). This pattern suggests that, in the absence of lesions, zinc may be more evenly distributed and present in higher concentrations in specific regions, reflecting its role in balancing bone metabolism and maintaining the integrity of healthy tissue (Yamaguchi, 2010). On the other hand, in the induced periapical lesions, the reduction in areas of high concentration may be associated with increased osteoclastic activity or the redistribution of the metal in response to the inflammatory and infectious process (Ceylan et al., 2021).

The potential advancements provided by the incorporation of strontium, cobalt and manganese into the composition of calcium phosphate bone cements are already established (Bernhardt et al., 2017; Cummings et al., 2017; Wu et al., 2020). Zinc‐releasing ceramics have been investigated due to the combination of their osteoinductive and immunomodulatory properties, which act synergistically (Huang et al., 2021). These strategies can enrich these materials with the biological properties specific to the elements, whilst modulating their physicochemical characteristics, a modification that could promote greater viability and activity of human mesenchymal stem cells and stimulate angiogenesis, highlighting the biological benefits associated with ionic modification (Montesi et al., 2017; Tadier et al., 2012; Thormann et al., 2013).

Although this study has limitations due to the use of an animal model, it provides preliminary data that can be expanded in clinical studies. A deeper understanding of the interactions of the metals investigated could open new perspectives for therapeutic strategies targeting bone formation and repair. Potential approaches include mineral replacement or chelation in the periapical microenvironment and local interventions to regulate metal levels, control inflammation and modulate oxidative stress.

The results indicate significant differences in mineral composition between periapical lesions and healthy tissues, leading to the rejection of the initial null hypothesis. A higher metallic content was observed in periapical lesions compared to sham controls, regardless of the metal analysed. These changes may be linked to disruptions in mineral homeostasis, which is crucial for bone tissue integrity, and could be associated with chronic inflammatory conditions such as apical periodontitis.

## CONCLUSION

This study demonstrated differences in the levels of various essential elements between conditions with periapical lesions and healthy controls. Significant changes were observed in calcium, iron, manganese, copper, sodium, potassium and magnesium levels, indicating that periapical lesions can directly affect the mineral homeostasis of periapical bone tissue. These findings emphasize the importance of investigating the mineral profile in bone lesions to gain a deeper understanding of the mechanisms involved in their pathophysiology and potentially aid in developing targeted therapeutic strategies.

## AUTHOR CONTRIBUTIONS

**Jennifer Santos Pereira:** Conceptualization, methodology, investigation and writing—original draft. **Brenda Fornazaro Moraes:** Investigation and data curation. **Anna Carolina Neves Leutz:** Investigation and resources. **Hellen Carolliny de Souza Nicolau:** Investigation and data curation. **Rafaela Caires Santos:** Data curation and investigation. **Talita Tartari:** Writing—review and editing, and validation. **Brenda Paula Figueiredo de Almeida Gomes:** Writing—review and editing, and validation. **Adriana de Jesus Soares:** Writing—review and editing, and validation. **Ana Cristina Padilha Janini:** Data curation, investigation and formal analysis, and validation. **Lauter Eston Pelepenko:** Supervision, conceptualization, methodology, data curation and writing. **Marina Angélica Marciano:** Conceptualization, supervision, project administration, and writing—review and editing.

## FUNDING INFORMATION

This study was funded by the Coordination for the Improvement of Higher Education Personnel (Capes): 001 and the São Paulo Research Foundation FAPESP 2022/03093‐9.

## CONFLICT OF INTEREST STATEMENT

The authors have no competing interests to declare regarding this study.

## ETHICS STATEMENT

The study was conducted following ethical guidelines approved by the Animal Use Ethics Committee under Protocol CEUA: 6219‐1/2023.

## Supporting information


Data S1

## Data Availability

The data that support the findings of this study are available from the corresponding author upon reasonable request.
