# Supplementary material for: Metallomic characterization of induced periapical lesions—In vivo study
Source: Int Endod J. 2025 Jul 1;58(10):1594–615. doi: 10.1111/iej.14274 (PMC12423490; doi:10.1111/iej.14274)
Supplement: Supplementary file 1 — Data S1 [file IEJ-58-1594-s001.docx]

Supplementary material for:

Metallomic characterisation of induced periapical lesions – in vivo study

Supplementary material 1 – Sample size calculation

Supplementary table 1 – Description of the reference methodologies used for sample size calculation, unit, estimated standard deviation, estimated minimum difference to be detected and estimated number of animals.

| Reference method | Unit | Estimated standard deviation | Estimated minimum detectable difference | N estimated / Observations |
| --- | --- | --- | --- | --- |
| Histological Analysis (Marciano et al., 2016) | Inflammatory score (0 to 3) | 0.5 average score | 1 | 10 |
| ICP-MS of relevant chemical elements adapted from literature studies (Grassin-Delyle et al., 2019; Marciano et al., 2023) | Mass fractions (ng/g) | 10 ng/g | 20 ng/g | 10 |
| Weight variation of animals (Marciano et al., 2023) | Grams (g) | 31.8 g (obtained from all animals before the procedure) | 18 g | 118 animals in total; however, reducible (3Rs) if normally distributed at baseline since this is not the main outcome of the study. |

**Supplementary material 2 – Experimental study design**


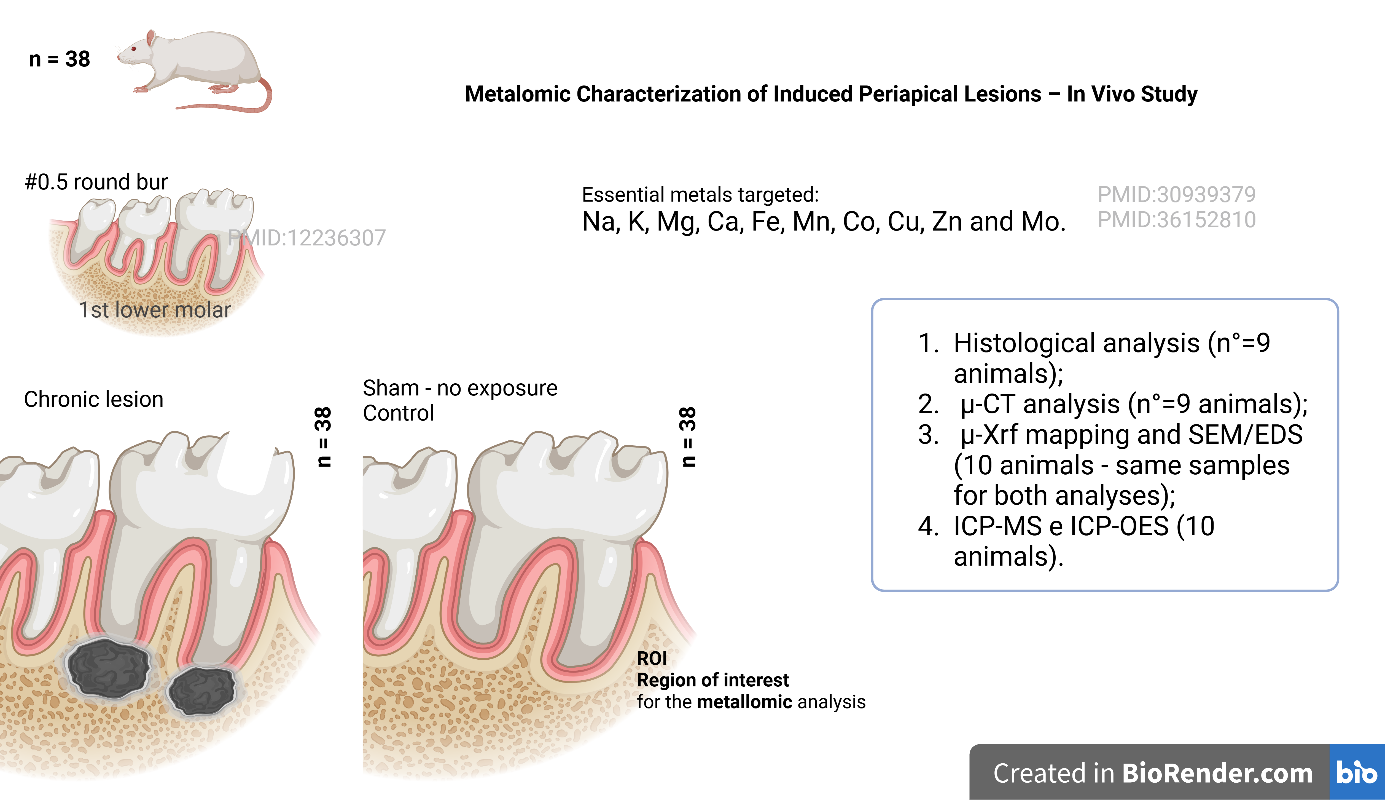


Figure S1: Experimental Scheme: Half of the animals underwent periapical lesion induction for 40 days, following previously proposed methodologies (Metzger et al., 2002; Gomes et al., 2019). For the sham control, identical procedures were performed, except for the lesion induction cavity in the first lower molars. During the immediate postoperative period, one animal designated for each experimental condition did not survive anaesthesia administration, leading to their exclusion from the study. As a result, the initial sample of 40 animals was reduced to 38, with 19 animals per group. After the 40-day induction period, all animals were euthanised, and different methodologies, including periapical radiography, X-ray Fluorescence Microscopy, histology, µ-CT, scanning electron microscopy with energy dispersive spectroscopy, ICP-MS, and ICP-OES, were conducted to establish the metallographic profile, focusing on the ten chemical elements considered essential for metabolism: sodium, potassium, magnesium, calcium, iron, manganese, cobalt, copper, zinc, and molybdenum, comparing the conditions with and without lesion in the region-of-interest (ROI).

Supplementary material 3 - Experimental sampling and storage scheme


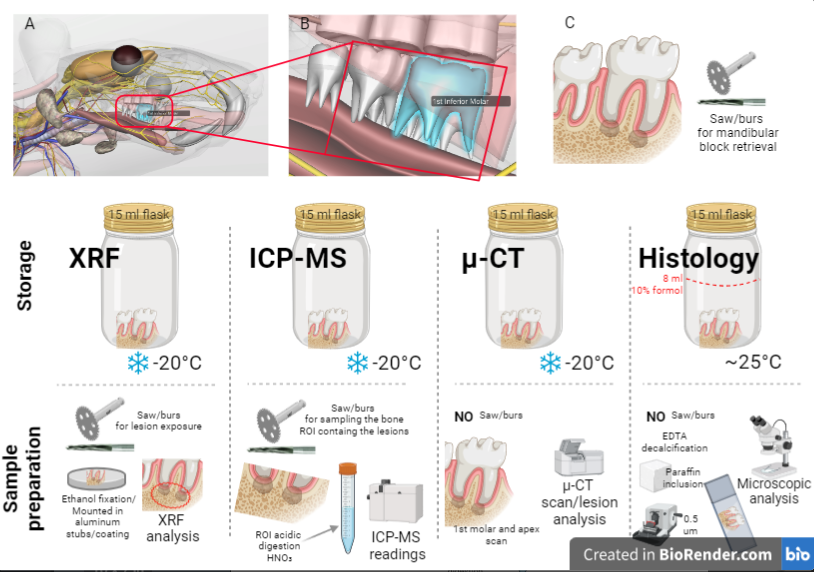


**Figure S2**: Experimental sampling and storage scheme: (A, B) Tissue that was removed from the animal's mandible (C) using blunt-ended scissors. Each sample was stored individually in 15 mL containers, following the specific methodology employed. The samples intended for analysis by µ-XRF, ICP-MS, and µ-CT were frozen at -20°C until processing. For histological analysis, the specimens were immersed in 8 mL of 10% formalin and stored protected from light at controlled room temperature. Before performing any method, all hemimandibles were subjected to digital radiographs. The preparation for µ-XRF involved exposing the lesion region, keeping it attached to the dental element, fixation, and dehydration in an increasing ethanol gradient, followed by mounting on stubs. For SEM/EDS analysis, carbon coating was applied for lesion analysis. For ICP-MS and ICP-OES analysis, the bone sample was separated from the dental element and digested in nitric acid under a microwave-activated pressure system to allow for the reading of the chemical elements of interest. For µ-CT analysis, no additional sectioning was performed, and the specimens were scanned, reconstructed, and analysed using specific software. For histological analysis, decalcification, paraffin embedding was performed to enable the use of a microtome (slices of 0.5 µm), which were then mounted and stained with H&E for analysis under an optical microscope.

Supplementary material 4 – µ-CT analysis


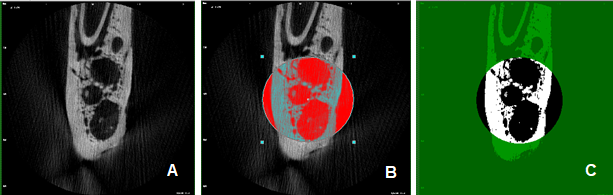


# **Figure S3**: (A) µ-CT image of the region of the first lower molar, axial view, with white arrow indicating the periapical lesion site adjacent to the mesial root. (B) Delimitation of the region of interest containing the induced periapical lesion (red circle). (C) Selected region of interest for analysis of the volume of the induced periapical lesion.

Supplementary material 5 - Operational conditions for ICPs analysis

**Supplementary table 2**: Settings and flow parameters for ICP-MS analysis

| Category | Parameter | Value |
| --- | --- | --- |
| Flows (L/min) | Plasma Flow | 9.0 |
|  | Auxiliary Flow | 1.5 |
|  | Protection Gas Flow | 0.0 |
|  | Nebulizer Flow | 1.03 |
| Torch Configuration | Sampling Depth (mm) | 5.0 |
| Operation | RF Power (kW) | 1.35 |
|  | Pump Rate (rpm) | 25 |
|  | Stabilization Time (s) | 15 |
| Skimmer and Gases | Skimmer Gas Source | He |
|  | Skimmer Flow (mL/min) | 80 |
|  | Nitrox - Flow (mL/min) | 0 |
|  | Skimmer - Polarization | 0.5 |

**Supplementary table 3**. Operational conditions of ICP-OES

| Parameter | Valor |
| --- | --- |
| Power | 1.450 W |
| Plasma Gas Flow | Not applicable |
| Auxiliary Gas Flow | 0.5 L/min |
| Nebulizer Gas Flow | 0.75 L/min |
| Nebulization Chamber | Cyclonic |

**Supplementary material 6 – Weight assessment
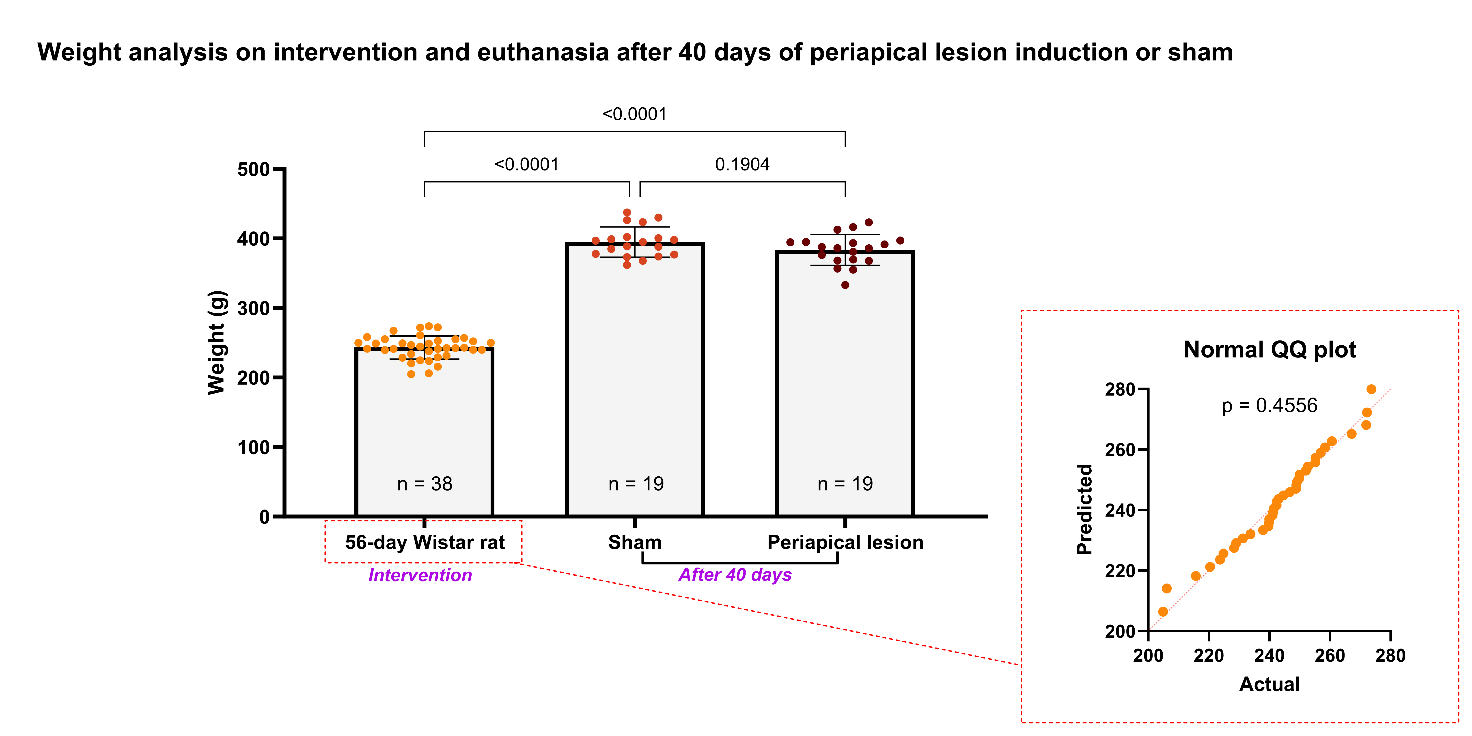
**

Figure S4: During the immediate postoperative period, one animal designated for each experimental condition did not survive anaesthesia administration, leading to their exclusion from the study. As a result, the initial sample of 40 animals was reduced to 38, with 19 animals per condition. The initial body weight of these 38 animals, recorded on the day of lesion induction (or its sham), followed a normal distribution, as indicated in the Normal QQ plot representation (p = 0.4556). After the 40-day experimental period, similar (p = 0.1904) body weight was observed between the two experimental conditions.

Supplementary material 7 – Radiographic images from all molars’ periapical region

#
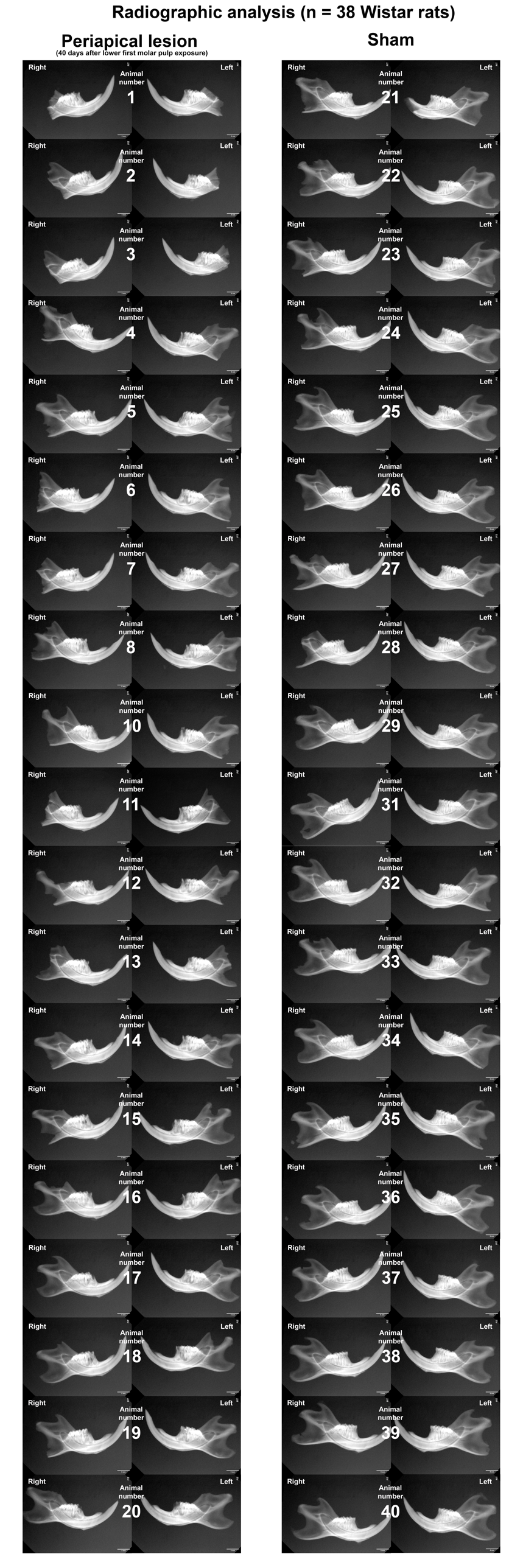


Figure S5: Periapical radiographs of Wistar rat (n = 38) hemimandibles (n = 76) obtained for the evaluation of bone structure within the two experimental conditions. The periapical lesions (left) underwent pulp exposure of the first lower molar for 40 days, leading to the induction of periapical lesions as suggested by the images; while sham controls (right) received no pulp exposure. Images were acquired using a high-resolution digital radiographic (SPECTRO 70X model and SOPRO – FIT Digital Sensor Software, Brazil) system with standardised exposure parameters to ensure comparability. Differences in radiopacity and bone continuity between conditions can be observed, allowing the identification of bone resorption areas associated with the periapical inflammatory process.

Reference list for this Supplementary material

Gomes, E.S.B., Farias, L.C., Silveira, L.H., Jesus, C.Í.D., Rocha, R.G.D., Ramos, G.V., Magalhães, H.T.A.T., Brito-Júnior, M., Santos, S.H.S., Jham, B.C., De Paula, A.M.B. & Guimarães, A.L.S. 2019. Conditioned fear stress increases bone resorption in apical periodontitislesions in Wistar male rats. *Archives of Oral Biology*, 97: 35–41. https://linkinghub.elsevier.com/retrieve/pii/S0003996918303662 11 December 2023.

Grassin-Delyle, S., Martin, M., Hamzaoui, O., Lamy, E., Jayle, C., Sage, E., Etting, I., Devillier, P. & Alvarez, J.-C. 2019. A high-resolution ICP-MS method for the determination of 38 inorganic elements in human whole blood, urine, hair and tissues after microwave digestion. *Talanta*, 199: 228–237. https://linkinghub.elsevier.com/retrieve/pii/S0039914019302115 27 March 2024.

Marciano, M.A., Guimarães, B.M., Amoroso-Silva, P., Camilleri, J. & Hungaro Duarte, M.A. 2016. Physical and Chemical Properties and Subcutaneous Implantation of Mineral Trioxide Aggregate Mixed with Propylene Glycol. *Journal of Endodontics*, 42(3): 474–479. https://linkinghub.elsevier.com/retrieve/pii/S0099239915009619 26 February 2024.

Marciano, M.A., Pelepenko, L.E., Francati, T.M., Antunes, T.B.M., Janini, A.C.P., Rohwedder, J.J.R., Shelton, R.M. & Camilleri, J. 2023. Bismuth release from endodontic materials: in vivo analysis using Wistar rats. *Scientific Reports*, 13(1): 9738. https://www.nature.com/articles/s41598-023-36690-4 28 December 2023.

Metzger, Z., Klein, H., Klein, A. & Tagger, M. 2002. Periapical Lesion Development in Rats Inhibited by Dexamethasone. *Journal of Endodontics*, 28(9): 643–645. http://linkinghub.elsevier.com/retrieve/pii/S0099239905604505 11 December 2023.
